# Activated-PAK4 predicts worse prognosis in breast cancer and promotes tumorigenesis through activation of PI3K/AKT signaling

**DOI:** 10.18632/oncotarget.7466

**Published:** 2016-02-17

**Authors:** Li-Fang He, Hong-Wu Xu, Min Chen, Zhi-Rong Xian, Xiao-Fen Wen, Min-Na Chen, Cai-Wen Du, Wen-He Huang, Jun-Dong Wu, Guo-Jun Zhang

**Affiliations:** ^1^ The Breast Center, Cancer Hospital of Shantou University Medical College, Shantou 515041, Guangdong, China; ^2^ Changjiang Scholar Laboratory, Shantou University Medical College, Shantou 515041, Guangdong, China; ^3^ Department of Neurosurgery, Second Affiliated Hospital of Shantou, University Medical College, Shantou 515031, Guangdong, China; ^4^ Department of Breast Medical Oncology, Cancer Hospital of Shantou University Medical College, Shantou 515041, Guangdong, China

**Keywords:** p21-activated kinase 4 (PAK4), prognostic marker, breast cancer, PI3K, AKT

## Abstract

The p21-activated kinase 4 (PAK4) is sufficient to transform noncancerous mammary epithelial cells and to form tumors in the mammary glands of mice. The accumulated information suggests that PAK4 might be an oncogenic protein in breast cancer. In this study, we sought to identify the role for PAK4 in breast cancer progression. Immunohistochemical study revealed that high PAK4 expression is associated with larger tumor size, lymph node metastasis, and advanced stage cancer in 93 invasive breast carcinoma patients. Moreover, high PAK4 expression was significantly associated with poor overall and disease-free survival. PAK4 remained an independent adverse prognosticator after univariate and multivariate analysis. Ectopic expression of wild-type PAK4 in MDA-MB-231 cells activated PI3K/AKT signaling and resulted in the enhancement of the cell proliferation, migration, and invasion, whereas PAK4-induced effects were blocked by the PAK4 kinase inhibitor PF- 3758309, PAK4 siRNAs or the PI3K inhibitor LY294002. Furthermore, a kinase-active PAK4 (S474E) strongly induced PI3K/AKT activation, and promoted proliferation, migration and invasion in breast cancer cells. A kinase-inactive PAK4 KD (K350A/K351A) did partially upregulate PI3K/AKT, and promoted invasive phenotype. Taken together, these findings suggest that PAK4-activated PI3K/AKT signaling is both kinase-dependent and -independent, which contributes to breast cancer progression. Thus, our results imply that dual inhibition of PAK4 and PI3K/AKT signaling might be a potential therapeutic approach for breast cancer therapy.

## INTRODUCTION

Breast cancer (BC) is one of the most commonly detected cancers and the second leading cause of cancer-related mortality affecting women worldwide [1]. The major reason for breast cancer-related deaths is metastasis. Identification of novel prognostic markers and a thorough understanding of the marker's mechanism could provide a basis for designing future therapeutic strategies for breast cancer patients.

The serine/threonine kinase PAK family members are activated in various human cancers (reviewed in refs. [2] and [3]). PAK4 is essential for embryonic viability and tissue development, but is not expressed at significant levels in the majority of normal adult tissues [4, 5]. *PAK4* gene amplification has been most frequently observed in multiple cancer such as oral squamous-cell carcinoma, pancreatic cancer and colon cancer [6–9], and is associated with aggressive disease and poor prognosis in oral squamous-cell carcinoma [7]. High PAK4 activity associated with poor prognosis in ovarian cancer patients [10]. High PAK4 activity is linked with many hallmarks of tumorigenesis, including anchorage independent growth [11–13], cell survival [14, 15], cell migration and invasion [6, 10, 16–19]. Moreover, elevated expression of PAK4 promotes cancer progression *in vitro* and *in vivo* [13, 16, 20, 21]. Previous studies on the mechanism of PAK4-enhanced tumor progression addressed several downstream effectors, such as c-Src, MEK-1/ERK1/2, MMP2, and c-Src/EGFR in ovarian cancer [10], p57^Kip2^ [22] and integrin αvβ5 [23] in breast cancer.

Numerous studies point to a striking role for PAK4 in breast cancer [24, 25]. PAK4 is barely detectable in normal epithelial tissue [20], but is frequently activated in breast cancer cell lines [13, 20, 26] as well as in primary human breast cancer specimens and rat mammary tumor samples [20]. High levels of PAK4 expression have been suggested to be a driving force for immortalization and tumorigenicity in mouse and human mammary epithelial cells [26, 27]. PAK4 overexpression also enhances the oncogenic phenotype [28] and cell migration in MDA-MB-231 cells [23]. Additionally, *PAK4* gene is located at chromosome 19q13.2, a region frequently amplified in aggressive breast cancers with basal-like features [29].

In the present study, we investigated whether PAK4 could be used as a biomarker of breast cancer progression and prognosis. Herein, we analyzed the PAK4 expression level in tumor tissue from 93 breast cancer patients, and also evaluated the association of PAK4 with clinical pathological parameters and patient survival. The role of PAK4 for cell migration, invasion, and proliferation was investigated in MBA-MB-231 cells either by overexpressing PAK4 or silencing PAK4 with RNAi, followed by a series of *in vitro* functional assays. Moreover, mechanistic studies revealed the involvement of PAK4–activated PI3K/AKT signaling contributes to PAK4-induced invasive phenotype. The present study supports the functional role of PAK4 in breast cancer, and suggests that PAK4 and PI3K/AKT signaling could serve as a novel target for breast cancer therapy.

## RESULTS

### Correlation of PAK4 expression with clinicopathological characteristics

**Figure 1:**
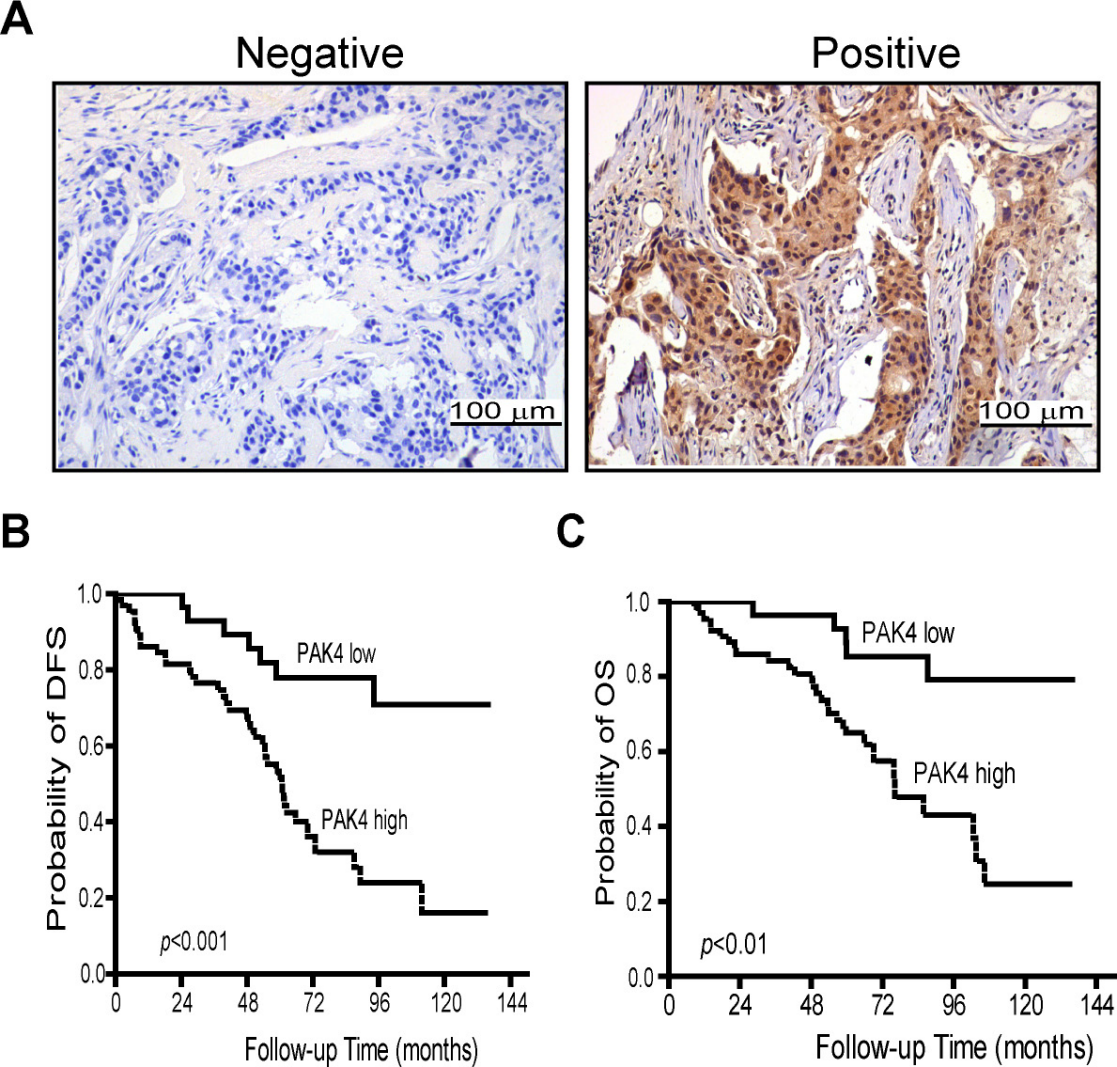
High-expression of PAK4 is associated with poor prognosis in breast cancer patients (**A**) Expression level of PAK4 was examined by immunohistochemistry in tumor samples from breast cancer patients. (**B** and **C**) Correlation of PAK4 expression with disease-free survival (DFS) and overall survival (OS). Patients in the PAK4-high expression group showed shorter survival and worse prognosis than the patients with in the PAK4-low expression group.

We analyzed the PAK4 expression pattern in 93 BC tissue specimens by immunohistochemistry. A total of 69.2% of BC specimens were positive for PAK4 immunostaining. Both cytoplasmic and nuclear staining of PAK4 was seen in tumor cells (Figure 1A). High level PAK4 expression was associated with more lymph node metastasis (*p* = 0.027), larger tumor size (*p* = 0.007), and advanced AJCC stages (*p* = 0.012) (Table 1). However, there was no association between PAK4 expression and age, histological grade, molecular classification based on HER2 (ErbB2), estrogen receptor (ER) or progesterone receptor (PR) status (Table 1).

**Table 1:** Association of PAK4 expression with clinicopathological parameters in breast cancer

	No. (%)	PAK4 expression		
		NR(%)	PR(%)	*p* value
Age				
≤50 y	51 (54.8)	12 (23.5)	39 (76.5)	0.128
>50 y	42 (45.2)	16 (38.1)	26 (61.9)	
Tumor size				
≤2 cm	10 (10.8)	7 (70.0)	3 (30.0)	0.007
>2 cm	83 (89.2)	21 (25.3)	62 (74.7)	
No. of lymph node metastasis				
≤3	61 (65.6)	23 (37.7)	38 (62.3)	0.027
>3	32 (34.4)	5 (15.6)	27 (84.4)	
AJCC TNM stage				
I+II	55 (59.1)	22 (40.0)	33 (60.0)	0.012
III	38 (40.9)	6 (15.8)	32 (84.2)	
Histological Grade				
1+2	51 (54.8)	18 (35.3)	33 (64.7)	0.230
3	42 (45.2)	10 (23.8)	32 (76.2)	
Molecular classification				
Luminal A (ER+/PR+/HER2-)	36 (38.7)	14 (38.9)	22 (61.1)	0.377
Luminal B (ER+/PR+/HER2+)	23 (24.7)	4 (17.4)	19 (82.6)	
HER2 enriched (ER-/PR-/HER2+)	17 (18.2)	5 (29.4)	12 (70.6)	
TNBC (ER-/PR-/HER2-)	17 (18.2)	5 (29.4)	12 (70.6)	

Abbreviations: NR, negative rate; PR, positive rate; AJCC, American Joint Committee on Cancer; ER, estrogen receptor; PR, progesterone receptor; TNBC, triple negative breast cancer.

### Univariate and multivariate analyses of PAK4 and prognostic variables in breast cancer patients

We performed Kaplan–Meier analyses to determine whether PAK4 expression was associated with disease-free survival (DFS) and overall survival (OS) for BC patients. Patients whose primary tumors expressed PAK4 had shorter DFS (median equal 60.7 months, *n* = 65) than those in the PAK4 negative group (median age greater > 144 months, *n* = 28) (*p* = 0.000) (Figure 1C). A statistically significant association of PAK4 with shorter OS was also found (*p* = 0.001) (Figure 1B). To further evaluate the prognostic value of PAK4 in breast cancer, we performed univariate and multivariate analysis on patient data. The relationships between DFS/OS and nine parameters including tumor size, lymph node status, histological grade, and AJCC stage, and expression of ER, PR, HER2, and PAK4 were assessed (Tables 2 and 3). In the univariate analysis, patients expressing high levels of PAK4 were specifically associated with poor disease-free or overall survival (HR = 4.1, 95% CI 1.819–9.251, *p* = 0.001 and HR = 4.249, 95% CI 1.622–11.135, *p* = 0.003, respectively, Table 2 and 3), as well as lymph node status (DFS, *p* = 0.000 and OS, *p* = 0.003, Table 2 and 3). In addition, tumor size was found to be associated only with DFS (*p* = 0.026, Table 2). No other significant correlation was found between survival and the other parameters including AJCC stage, or expression of ER, PR, or HER2. When the nine parameters were included in the Cox multivariate analysis, PAK4 and lymph node status stood out as independent prognostic factors with regard to both DFS (HR = 2.9, *p* = 0.011; HR = 3.1, *p* = 0.000 respectively, Table 2) and OS (HR = 3.713, *p* = 0.009; HR = 2.385, *p* = 0.017, respectively, Table 3), and tumor size was an independent prognostic factor with regard to DFS (HR = 0.116, *p* = 0.035, Table 2).

**Table 2:** Univariate and multivariate analysis for the effect of PAK4 on disease-free survival in breast cancer patients

	Univariate Analysis			Multivariate Analysis		
	HR	95% CI	*p* value	HR	95% Cl	*p* value
PAK4, + vs −	4.100	1.819–9.251	0.001	2.904	1.277–6.605	0.011
NLNM, > 3 vs < 3	3.109	1.731–5.582	0.000	3.315	1.714–5.734	0.000
TS, > 2 cm vs < 2 cm	0.105	0.014–0.767	0.026	0.116	0.016–0.859	0.035
HG, 3 vs 1 + 2	1.530	0.861–2.720	0.147			

Abbreviations: HR, Hazard Ratio; CI, confidence Interval; NLNM, No. of lymph node metastasis; TS, Tumor Size; HG, Histological Grade.

**Table 3:** Univariate and multivariate analysis for the effect of PAK4 on overall survival in breast cancer patients

	Univariate Analysis			Multivariate Analysis		
	HR	95% CI	*p* value	HR	95% Cl	*p* value
PAK4, + vs −	4.249	1.622–11.100	0.003	3.713	1.395–9.879	0.009
NLNM, > 3 vs < 3	2.852	1.414–5.750	0.003	2.385	1.167–4.872	0.017
TS, > 2 cm vs < 2 cm	0.163	0.022–1.195	0.074			
HG, 3 vs 1 + 2	2.066	1.039–4.108	0.039			

Abbreviations: HR, Hazard Ratio; CI, confidence Interval; NLNM, No. of lymph node metastasis; TS, Tumor Size; HG, Histological Grade.

### Overexpression of PAK4 promotes breast cancer cell proliferation, migration, and invasion

**Figure 2:**
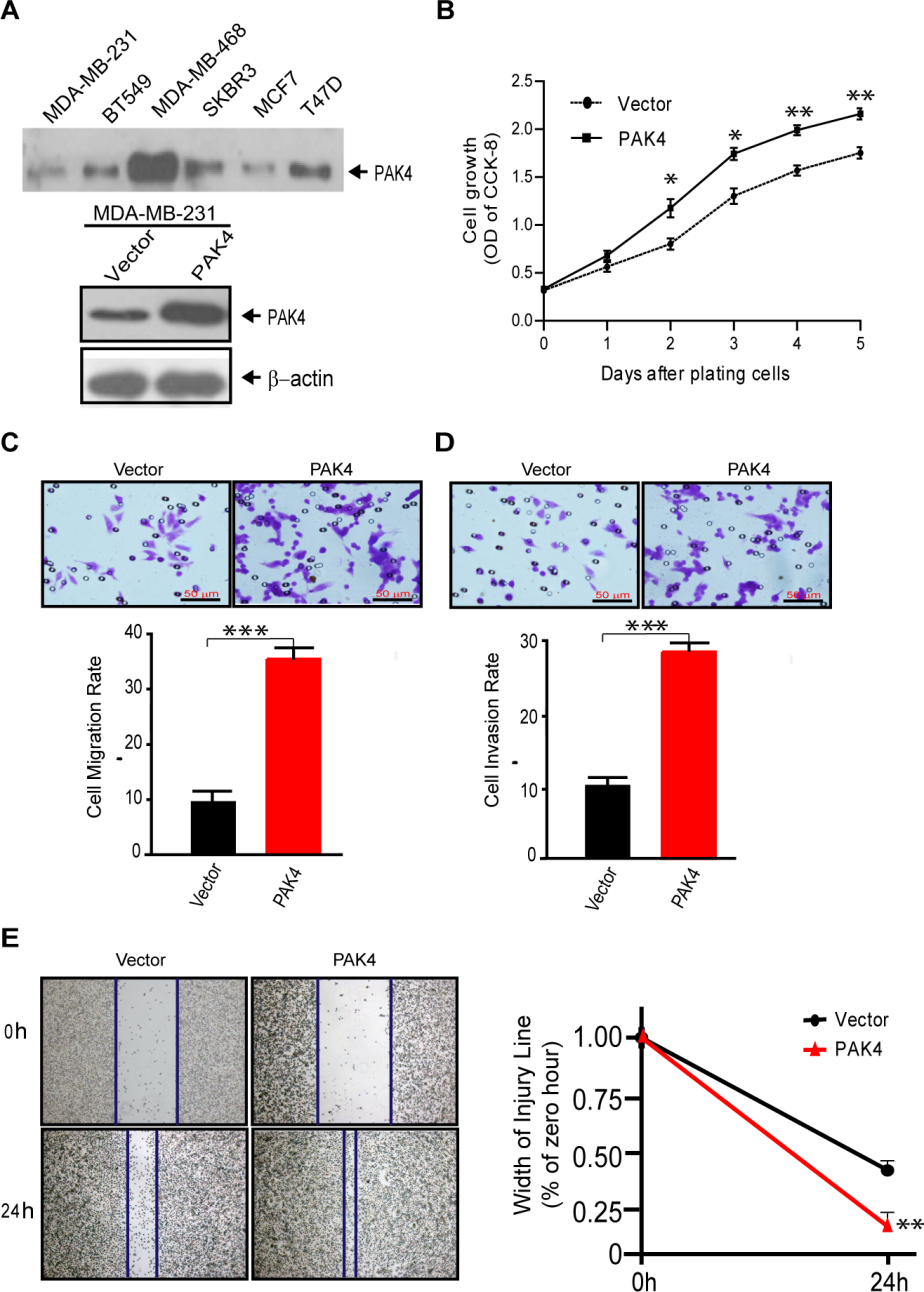
Overexpression of PAK4 increases the proliferation, migration, and invasion of breast cancer cells (**A**) Western blot analyses of PAK4 expression level in breast cancer cell lines (upper panel) and ectopic PAK4 expression in MDA-MB-231cells (lower panel). β-Actin was used as an internal control. (**B**) Cell growth was determined by CCK8 assay in ectopic PAK4 expressing cells, as well as control cells. Data represent the mean ± SD of 5 experiments. (**C**) Transwell migration assay and (**D**) invasion assay were used to analyze the effect of overexpressed PAK4 on cell motility. Data is presented as the mean ± SEM (*n* = 3). (**E**) The effect of PAK4 on cell motility was assessed by a wound-healing assay. Flag-tagged wild-type PAK4 plasmid or p3XFLAG-CMV-10 control expressing cells were grown in six-well plates for 48 hours, then scratched using sterile pipet tips and recovered in medium containing 10% fetal bovine serum. Phase contrast micrograph images were recorded at the time of wounding 0 and 24 h later. Width of injury line (% of zero hour) is presented as mean ± SEM (*n* = 3). *, ** and *** denote *p* < 0.05, *p* < 0.01 and *p* < 0.001.

We performed a series of functional assays to determine the effects of high level PAK4 on cell proliferation and invasion of breast cancer cells. First, we found that PAK4 over-expression (Figure 2A) enhanced MDA-MB-231 cell proliferation between 2 to 5 days after transfection (Figure 2B). Next, the rate of migration and invasion of breast cancer cells overexpressing PAK4 increased 3.5 fold and 2.88 fold, respectively, compared to control cells (Figure 2C and 2D, p < 0.001). PAK4 overexpression also promoted tumor cell migration in a wound healing assay (Figure 2E).

### PAK4 knockdown suppresses cell proliferation, migration and invasion

**Figure 3:**
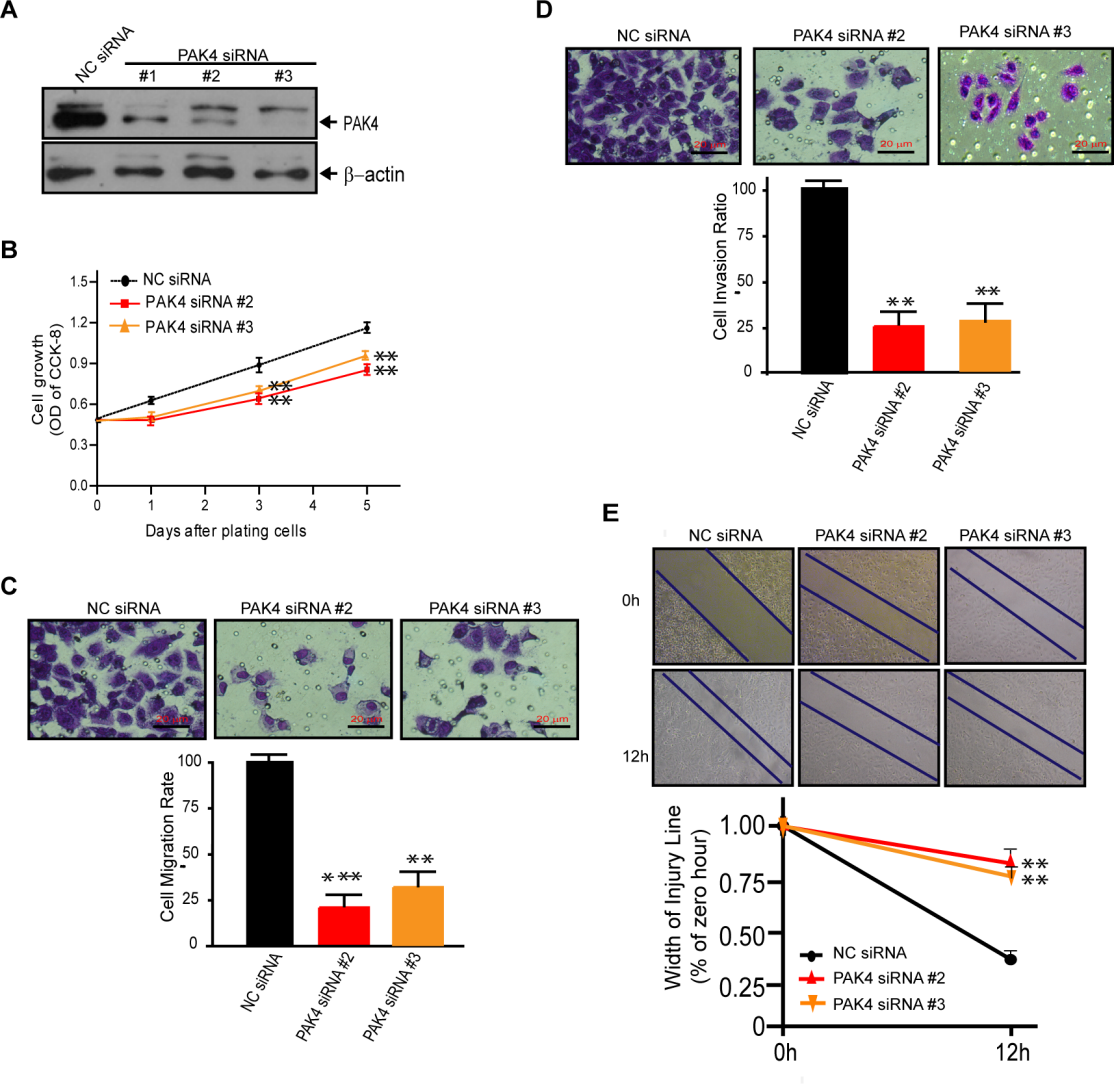
Knock-down of PAK4 by siRNAs suppresses cell proliferation, migration, and invasion of MDA-MB-231 cells (**A**) Western blot validating decreased PAK4 expression in MDA-MB-231 cells following transfection with PAK4 siRNAs versus a control NC siRNA. β-Actin was used as the internal control. (**B**) Inhibition of cell growth measured by CCK8 assay was determined by comparing PAK4-silenced cells to cells with NC siRNA. Data is presented as mean ± SD of 3 experiments. (**C**) Transwell migration assay and (**D**) invasion assay was used to analyze the effect of PAK4 siRNAs (#2 and #3) on breast cancer cell motility. Data is presented as the mean ± SEM (*n* = 3). (**E**) The effect of PAK4 siRNAs on cell motility was assessed by a wound-healing assay. Confluent monolayers of PAK4-silenced cells or control cells were grown in six-well cluster plates for 48 hours, scratched using sterile pipet tips and recovered in medium containing 10% fetal bovine serum. Phase contrast micrograph images were recorded at the time of wounding (0 h), and at 12 h. Width of the injury line (% of zero hour) is presented as the mean ± SEM (*n* = 3). *, ** and *** denote p < 0.05, *p* < 0.01 and *p* < 0.001.

To provide definitive proof of concept that PAK4 expression actively participates in breast tumorigenesis, we assessed the effects of PAK4 siRNA on cell proliferation and invasion in MDA-MB-231 cells. The expression of PAK4 protein was significantly knock-down by three individual PAK4 siRNAs (Figure 3A). When compared to control siRNA, selective PAK4 siRNAs #2 and #3, reduced cell growth by 19.6% and 19.4%, 28.4% and 27.9%, and 26.5% and 25.8% at 1, 3 and 5 days post transfection, respectively (Figure 3B). Moreover, the reduction of PAK4 expression led to a dramatic inhibition of tumor cell migration (Figure 3C, 4.26-fold PAK4 siRNA #2, *p* < 0.001; 3.88-fold PAK4 siRNA #3, *p* < 0.01) and invasion (Figure 3D, 4.08-fold PAK4 siRNA #2; 3.76-fold PAK4 siRNA #3, both *p* < 0.01). Additionally, wound healing assays showed PAK4 siRNA #2 or #3 significantly inhibited tumor cell migration (Figure 3E, *p* < 0.01).

### PAK4 upregulates PI3K expression and activates PI3K/AKT signaling

**Figure 4:**
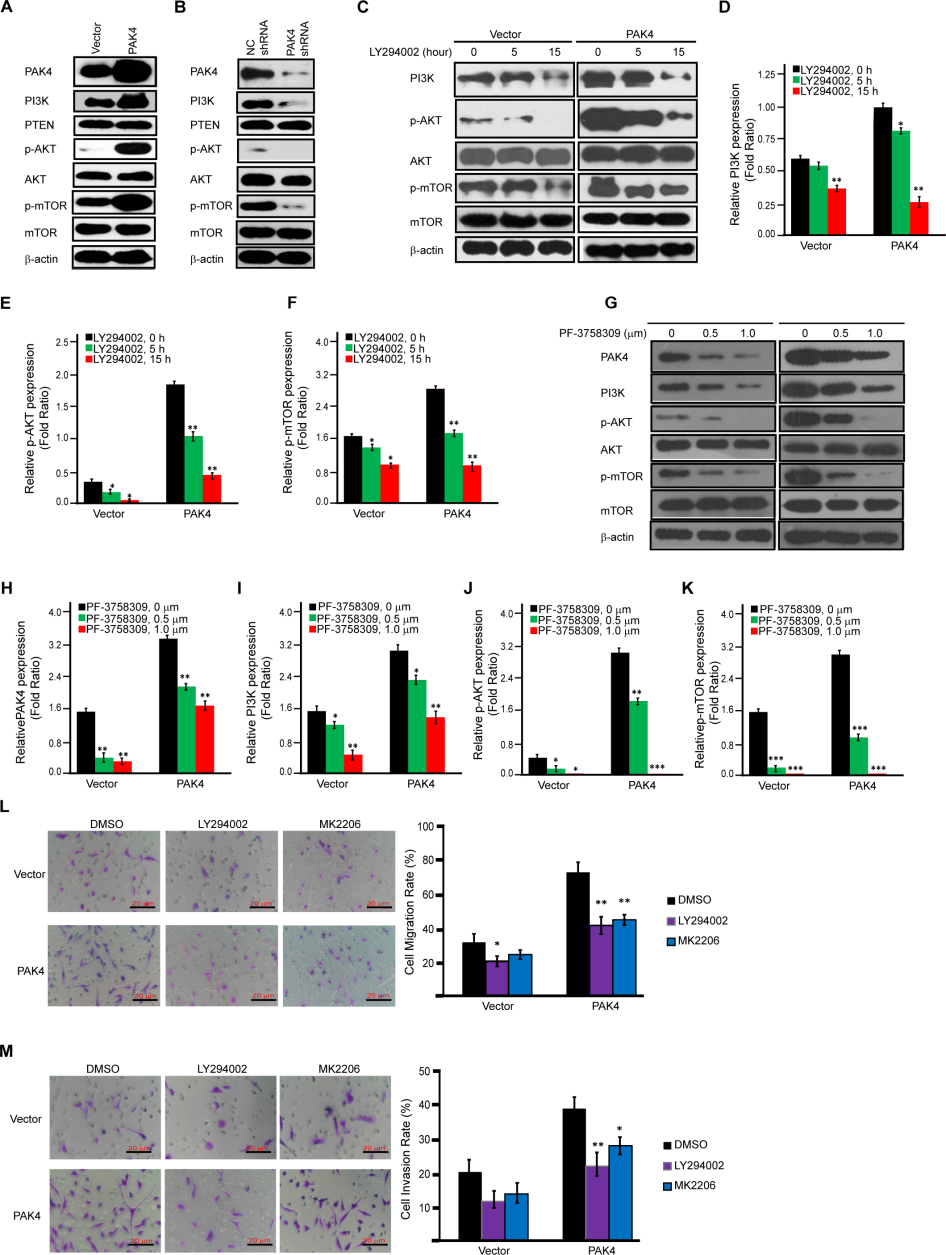
PAK4-activated PI3K/AKT signaling cascades in MDA-MB-231 cells (**A** and **B**) Western-blot analysis was used to determine the effect of PAK4 overexpression (A) or PAK4 siRNAs (B) on proteins involved in the AKT signaling cascade. (**C**–**F**), Effect of LY294002 on the expression level of proteins involved in PAK4-activated AKT signaling cascades. Immunoblot analysis of AKT signaling cascades after vector or PAK4-expressing MDA-MB-231 cells were treated with the PI3K inhibitor (LY294002, 20 μM) for 5 and 15 h (C). For densitometric analysis of PI3K, p-AKT and p-mTOR, immunoblots were normalized to β-actin (D–F). (**G**–**K**) Effect of PF-3758309 on the expression level of proteins involved in PAK4-activated AKT signaling cascades. Immunoblot analysis of AKT signaling cascades after 72 hour of treatment with two doses (0.5 or 1.0 μM) of PF-3758309 in vector or PAK4- expressing MDA-MB-231 cells (G). For densitometric analysis of PAK4, PI3K, p-AKT and p-mTOR, immunoblots were normalized to β-actin (H-K). (**L** and **M**) The effect of PI3K inhibitor (LY294002) and AKT signaling inhibitor (MK2206) on PAK4-induced cell migration and invasion. Transwell migration assay (L) and invasion assay (M) were performed after vector or PAK4-expressing MDA-MB-231 cells were treated with PI3K inhibitor (LY294002, 20 μM) for 15 h or AKT signaling inhibitor (MK2206, 30 μM) for 48 hour. Data is presented as the mean ± SD (*n* = 3). *, ** and *** denote *p* < 0.05, *p* < 0.01 and *p* < 0.001.

Since PI3K/AKT signaling has been found activated in NIH3T3 cells overexpressing PAK4 [13] and in pancreatic cancer cells [30], we attempted to determine the effect of PAK4 on PI3K/AKT signaling in breast cancer cells. Increased PI3K expression and phosphorylation of AKT and mTOR was observed in MDA-MB-231 cells overexpressing PAK4, while no effect was observed on the expression of total AKT and mTOR (Figure 4A). In contrast, PAK4 knockdown decreased PI3K levels and phosphorylation of both AKT and mTOR (Figure 4B). To confirm the importance of PI3K in PAK4-induced activation of p-AKT and p-mTOR, MDA-MB-231 cells were treated with LY294002, a pharmacological inhibitor of PI3K. Fifteen hours after LY294002 addition, PAK4-induced phosphorylation of AKT and mTOR was significantly reduced by 75.2% and 69.5%, respectively (Figure 4C–4F, both *p* < 0.01). PAK4-induced activation of the PI3K/AKT signaling was blocked by PF-3758309, an ATP-competitive inhibitor of PAK4 in dose-dependent manner (0.5 and 1.0 μM) (Figure 4G–4K). Moreover, both PI3K and AKT inhibitors (LY294002 and MK2206) blocked the PAK4-induced tumor cell migration and invasion (Figure 4L and 4M).

### PI3K/AKT signaling activation mediated by PAK4 is both kinase-dependent and -independent

**Figure 5:**
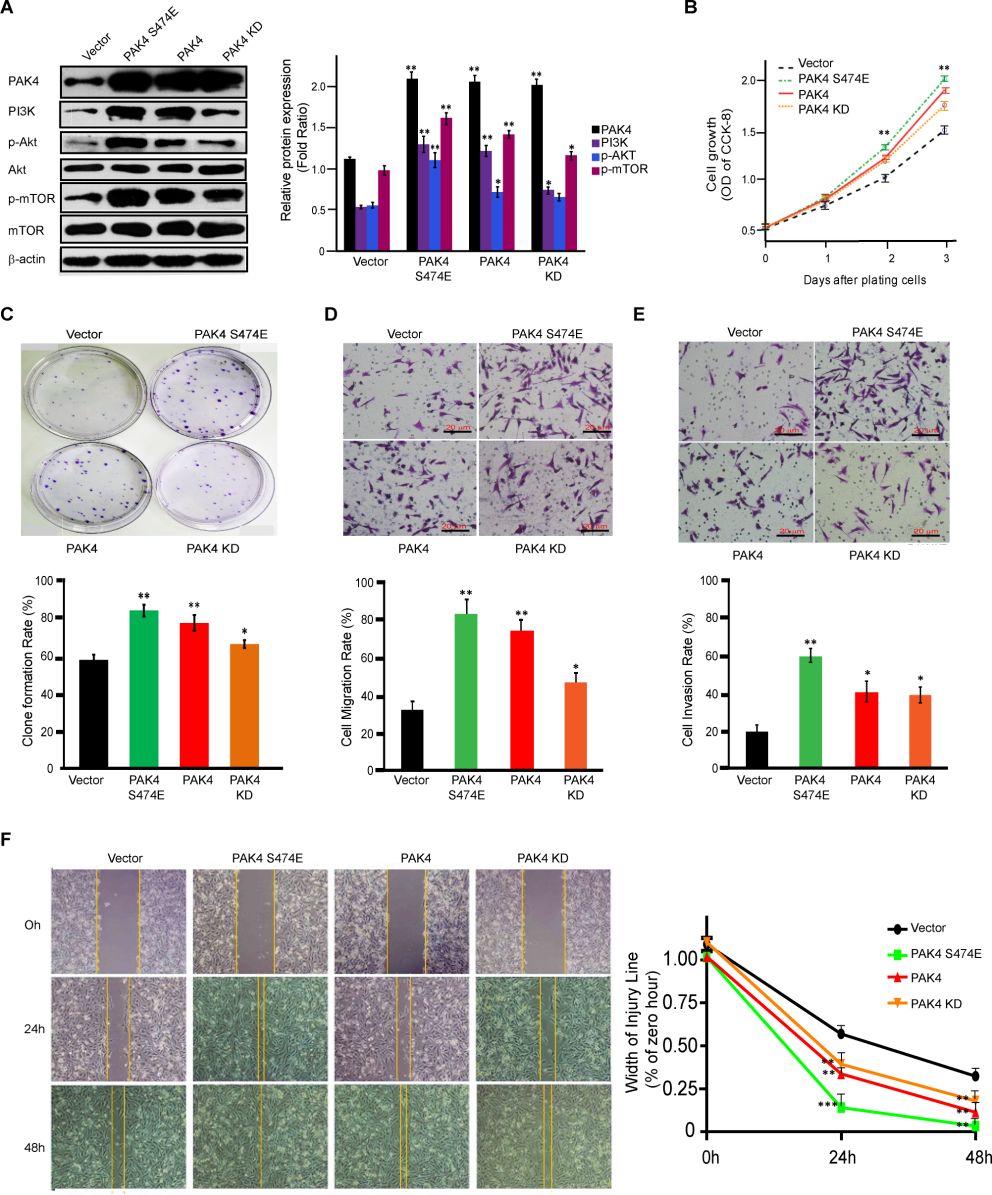
PAK4 promotes proliferation and invasive potential of MDA-MB-231 cells in both kinase-dependent and -independent manners (**A**) Western blot analysis was used to determine the effect of PAK4 wild-type and two kinase mutants, PAK4 (S474E) and PAK4 (K350A/K351A), on the activation of PI3K/AKT signaling. For densitometric analysis of PAK4, PI3K, p-AKT and p-mTOR, immunoblots were normalized to β-actin. (**B**) The effect on cell growth measured by CCK8 assay was determined by comparing PAK4-, PAK4 (S474E)- and PAK4 (K350A/K351A)-expressing cells to control vector cells. Data is presented as the mean ± SD of 3 experiments. (**C**) The effect of PAK4, PAK4 (S474E) and PAK4 (K350A/K351A) expression on cell clonality. Data is presented as the mean ± SD (*n* = 3). (**D**) Transwell migration, (**E**) invasion, and (**F**) wound-healing assays were used to analyze the effect of expressed PAK4, PAK4 (S474E), and PAK4 (K350A/K351A) on cell motility. Data is presented as the mean ± SD of 3 experiments. For wound healing, width of the injury line (% of zero hour) is presented as the mean ± SEM (*n* = 3). *, ** and *** denote *p* < 0.05, *p* < 0.01 and *p* < 0.001.

To further investigate the possible role of PAK4 kinase activity in PI3K/AKT activation, we analyzed whether constitutively active mutant of PAK4 or inactive mutant changes the regulation of PAK4-induced PI3K/AKT signaling. First, we found that PAK4 induced PI3K and the phosphorylation level of AKT and mTOR. In contrast to wild-type PAK4, a kinase-active PAK4 (S474E) enhanced PI3K/AKT signaling and a kinase-dead PAK4 KD (K350A/K351A) did partially upregulated PI3K/AKT (Figure 5A). Furthermore, among different PAK4 expression vectors, PAK4 (S474E) enhanced the cell proliferation (Figure 5B), clone formation (Figure 5C), migration (Figure 5D and 5F), and invasion (Figure 5E) at the most degree, followed by wild type PAK4 and PAK4 KD. Kinase-inactive PAK4 KD promoted invasive phenotype in comparison to control vector. Collectively, our results strongly indicated that PAK4 enhanced proliferation and invasive potential of MDA-MB-231 cells due to PAK4-activated PI3K/AKT signaling in both kinase-dependent and –independent manners.

### PAK4 increases tumor growth in mouse xenograft

**Figure 6:**
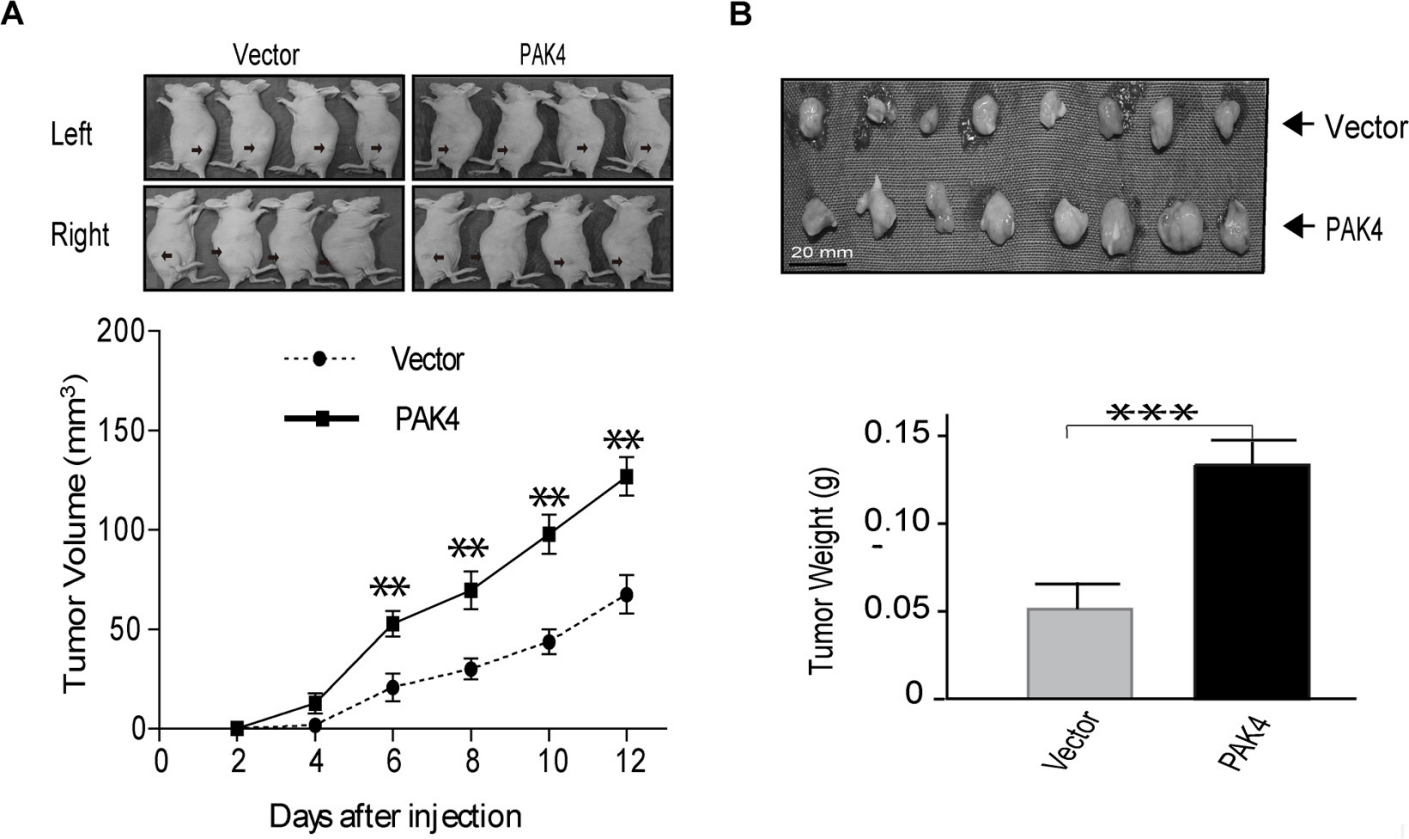
PAK4 promotes MDA-MB-231 breast xenograft tumor formation (**A**) Breast cancer cells transfected with PAK4 or control vector were implanted subcutaneously, at 2.5 × 10^6^ cells/mouse, into the flanks of nude mice. Tumors were observed and measured up to 12 days post implantation. Representative pictures of tumor nodules are presented. The growth curve was presented base on the average tumor volume. Bars represent SD. **p < 0.01. (**B**) At the 12 days after implantation, mice were euthanized and tumor nodules were excised, weighed, and presented as the mean ± SD. ***p < 0.001.

To determine whether high levels of PAK4 could promote tumor growth *in vivo*, MDA-MB-231 cells stably overexpressing PAK4 were implanted subcutaneously into the flanks of nude mice. The growth of tumor cells in mice injected with PAK4-expressing cells faster than control animals at different time points (Figure 6A). 12 days after xenograft, larger tumor nodules were frequently seen in PAK4-expressing tumor cells (Figure 6B), and the average tumor weight in PAK4 group was 2.44-fold larger than in control animals (Figure 6C). These results confirmed that high levels of PAK4 expression increased tumor growth in an MDA-MB-231 mouse xenograft model.

## DISCUSSION

This is the first comprehensive study to reveal the oncogenic and prognostic roles of PAK4 in breast cancer cells and cancer patients. Though previous studies have demonstrated that PAK4 expression is associated with aggressive oncogenic activity in breast cancer cells [13, 26–28], those studies had performed either in the breast cancer cell lines or mouse mammary gland tumor. Our results demonstrate that high level of PAK4 expression is associated with larger tumor size, more lymph node involved and advanced stages of cancer, and correlated with poor DFS and OS in breast cancer patients. Additionally, univariate and multivariate analyses identified that PAK4 is the most significant prognostic marker for breast cancer patients in addition to lymph node status.

We found that PAK4 activates PI3K/AKT signaling in the MDA-MB-231 breast cancer cell line, and identified a strong link between PAK4 induced invasive phenotype and PAK4 activated PI3K/AKT signaling. First, our *in vitro* and *in vivo* studies demonstrated that high level PAK4 expression induces breast cancer cell growth and promoted clone formation, cell migration and invasion, which is consistent with previous reports [23, 28]. We also identified that PI3K/AKT functions as a downstream of PAK4. Higher levels of phosphorylated AKT and PAK4 were previously found in tumorigenic cells compared to non-tumorigenic MCF10A mammary gland cells [27]. PAK4 and AKT signaling pathways can be reciprocally activated during chemotherapy [31, 32]. Our data illustrated that PI3K was the trigger for PAK4-induced AKT signaling. Exact molecular mechanism(s) involved in the regulation of PI3K by PAK4 needs further investigation. Importantly, PAK4-induced PI3K/AKT signaling contributes to PAK4 induced invasive phenotype.

The mechanisms for PAK4 promoting oncogenesis could be kinase–dependent [30, 31]. Previous finding that mutation at PAK4 Ser474 increases its catalytic activity [13, 33], whereas the K350A/K351A mutation had no detectable kinase activity [13]. In contrast to wild-type PAK4, PAK4 (S474E) induced more PI3K expression, much higher phosphorylated AKT and mTOR. Conversely, kinase-inactive mutant PAK4 KD (K350A/K351) resulted in partial induction of PI3K and phosphorylation of AKT and mTOR. Among PAK4 wild type and PAK4 kinase mutant vectors, constitutively active PAK4 (S474E) has the highest abilities for the induction of cell proliferation, clone formation, migration and invasion. This data proves that PAK4 induced invasive phenotypes dependent on PAK4 kinase activity. Alternatively, kinase-inactive PAK4 KD still significantly induced cell proliferation, clone formation, migration and invasion in contrast to control vector, and in some instance, functions as well as wild-type PAK4. Interestingly, PAK4 has been shown to promote cell survival and protect cells against apoptotic cell death through both kinase-dependent and kinase-independent mechanisms [14, 15, 34]. This is due to PAKs including PAK1 and PAK4 operate not only as protein kinases but also as scaffold proteins that mediate important protein-protein interactions [34–36]. This raises the possibility that PAK4 induced invasive phenotypes could be in both kinase-dependent and -independent manner.

This report first revealed the prognostic significance of PAK4 and its association with aggressive behavior in breast cancer. Our *in vitro* and *in vivo* results paralleled and supported the *in silico* data, demonstrated that PAK4 induced oncogenic features through the activation of PI3K/AKT pathway in both kinase-dependent and kinase-independent manner, suggesting dual inhibition for PAK4 and PI3K/AKT signaling could be a unique potential therapeutic approach for breast cancer therapy.

## MATERIALS AND METHODS

### Patients and tumor samples

A total of 93 paraffin embedded breast cancer specimens were collected from the Department of Breast Center between 2000 and 2006 (Shantou University Medical College). Clinical information for all BC patients with respect to age, tumor size, lymph node metastasis, tumor grade, estrogen and progesterone receptor, and HER2 status was collected. All patients had undergone surgery and were diagnosed with invasive ductal carcinoma with no evidence of metastasis at the first visit. The diagnosis of each sample was assessed by pathologists to ensure the sample was composed of more than 70% tumor cells. The use of human tissues in this study was approved by the Ethics Committee of Shantou University Medical College. This study was conducted according to the principles expressed in the Declaration of Helsinki.

### Immunohistochemistry

Briefly, formalin-fixed paraffin sections were stained with rabbit anti-PAK4 antibody by streptavidin-peroxidase. Epitope retrieval was achieved in a microwave by pretreatment with sodium citrate buffer, pH 6.0. Omission or replacement of the primary antibody with preimmune IgG serum was used as a negative control. Immunoreactivity was semi-quantitatively assessed by intensity and percentage of epithelium stained [10]. Staining intensity was scored as 0 (negative), 1 (faint), 2 (moderate), and 3 (strong). The percentage of positive staining was rated as 0 (< 5%), 1 (5–25%), 2 (26–50%), 3 (51–75%), and 4 (> 75%). A composite “histoscore” was given as a product of the average staining intensity (0–3) and average percentage of positive cells (0–4), with a maximum of 12. The level of PAK4 staining was calculated by adding the scores of staining intensity and the percentage of positive cells to define low-expression (0–6) and high-expression (7–12).

### Cell lines and plasmids

Human breast cancer cell lines MDA-MB-231, BT549, MDA-MB-468, SKBR3, MCF7, and T47D were purchased from the American Type Culture Collection. All cell lines were cultured in DMEM (GIBCO, Grand Island, USA) supplemented with 10% fetal calf serum (GIBCO, Grand Island, USA) at 37°C in an incubator with humidified atmosphere of 5% CO_2_ and 95% air. Flag-tagged wild-type PAK4 was a kind gift from Professor Staffan Strömblad (Center for Biosciences, Department of Biosciences and Nutrition, Karolinska Institutet, SE-141 83, Stockholm, Sweden) [37]. To facilitate mutagenesis, mutations were introduced by PCR and confirmed by sequencing of the complete cDNA fragment, which was then subcloned into the p3XFLAG-CMV-10 expression vector. The K350A and K351A mutation [13] was introduced with the oligo 5′-AGCTCGGGCAAGCTTGTGGCC GTCGCGGCCATG GACCTGCGCAAGCAGCAG-3′. The S474E mutation [13] was introduced with the oligo 5′-CAAGGAAGTGCCGCGGAGGAAGGAGCTGGTC GGCACGCCCTACTGGATG-3′.

### Compounds

LY294002 was purchased from Pfizer and dissolved in dimethyl sulfoxide (DMSO) as a 10-mmol/L stock. MK2206 was purchased from Selleck Chemicals and dissolved in dimethyl sulfoxide (DMSO) as a 10-mmol/L stock. PF-3758309 was purchased from Pfizer and dissolved in dimethyl sulfoxide (DMSO) as a 2-mmol/L stock.

### Generation of stable PAK4 overexpression and knock-down cell lines

To stably express PAK4, MDA-MB-231 cells were transfected with Flag-tagged wild-type PAK4 or p3XFLAG-CMV-10 (vector) using Lipofectamine 2000 (Invitrogen, California, USA) following the instructions provided by manufacturer and selected for with G418 (1000 ug/ml). To transiently knockdown PAK4, siRNA against PAK4 (#1: UGGUAAUCAUGAGGGACUATT, #2: GGAUGAACGAGGAGCAGAUTT and #3:GACUGAA GAACCUGCACAATT) or non-targeting control (NC) siRNA was transfected into MDA-MB-231cells for 48 h before cells were plated for proliferation, wound healing, migration, and invasion assays. Cells were harvested for total RNA and protein extraction after a 48 h transfection. To establish stable cell lines expressing PAK4 shRNA or non-targeting control (NC) shRNA, plasmids containing PAK4 shRNA or NC shRNA (Maji, Shanghai, China) were transfected into MDA-MB-231 cells and selected with G418.

### Immunoblotting

Cells were lysed with RIPA Lysis Buffer (Beyotime, Shanghai, China) and cleared by centrifugation at 4°C. Protein concentration was determined with a BCA Protein Assay Kit (Beyotime, Shanghai, China). A total of 30 μg protein was resolved by SDS/PAGE, transferred to polyvinylidene difluoride membrane, and probed with the corresponding antibodies: PAK4, p-AKT, AKT, m-TOR, p-mTOR, PI3K, PTEN (Cell Signaling Technology, Danvers, MA, USA) and β-actin (Santa Cruz Biotechnology, Santa Cruz, CA, USA).

### Cell viability assay

MDA-MB-231 cells (1 × 10^3^/well) were plated in 0.1 ml of the medium containing 10% FBS in 96-well plates. After addition of 10 μl of Cell Counting Kit-8 (Beyotime, Shanghai, China) to each well at 0, 1, 2, 3, 4, and 5 days after plating, cells were incubated for an additional 4 h. Absorbance was measured at 490 nm (BioTek Instruments, Inc, USA).

### Cellular migration and invasion assays

In total, 1 × 10^5^ MDA-MB-231 cells were added into the upper compartment of a transwell chamber (BD, Franklin Lakes, USA). Transwell inserts with an uncoated microporous (8 μm pore size) membrane were used for migration assays [10], in which 1 × 10^4^ MDA-MB-231 cells were added into the upper compartment of a transwell chamber. In invasion assay, BD BioCoat^™^ Matrigel^™^ Invasion Chamber was used. After 24-h or 48-h incubation, non-migrated or non-invaded cells remaining on the upper side of transwell inserts were cleared with a cotton swab. The migrated or invaded cells on the lower side of inserts were fixed, stained, and counted. All the experiments were conducted in triplicate.

### Wound healing assay

Cells were inoculated in 6-well plates at 48 h post-transfection, and were wounded with a sterile 200- μl pipette tip. Fresh culture medium was added. Phase contrast microscopic images were taken at the same position of the wound at time 0 and 12 h [21]. The width of the open areas was measured using Photoshop (Adobe), and the results averaged.

### Colony formation assay

Four hundred cells were inoculated into 6-well plates for growth and then stained with crystal violet for observation of colony formation after two weeks.

### Tumor xenografts

A total of 2.5 × 10^6^ MDA-MB-231 cells expressing either PAK4 or vector in 100 μl of PBS were s.c. injected into 5 female Nu/Nu mice. After implantation, diameters for tumors were measured perpendicularly on days 2, 4, 6, 8, 10, and 12, and volumes were calculated using caliper measurements with the equation: length × width^2^/2. Inoculation mice were euthanized at 12 days post injection, and tumor weight was determined [38]. All animal protocols were approved by the Ethics Committee of Shantou University Medical College.

### Statistical analysis

Either the chi-squared test or Fisher's exact test was performed to determine the correlation between PAK4 expression and its related proteins and clinical pathological features of breast cancer. Survival curves were estimated using the Kaplan-Meier method, and significant differences between survival curves were determined using a log-rank test. Cox regression analyses (univariate and multivariate analysis) were performed for DFS and OS, to assess the prognostic or predictive significance of the examined markers. Unless otherwise stated, all *P*-values were calculated by means of a two-sided *t*-test where *P*-values < 0.05 were considered as significant.
